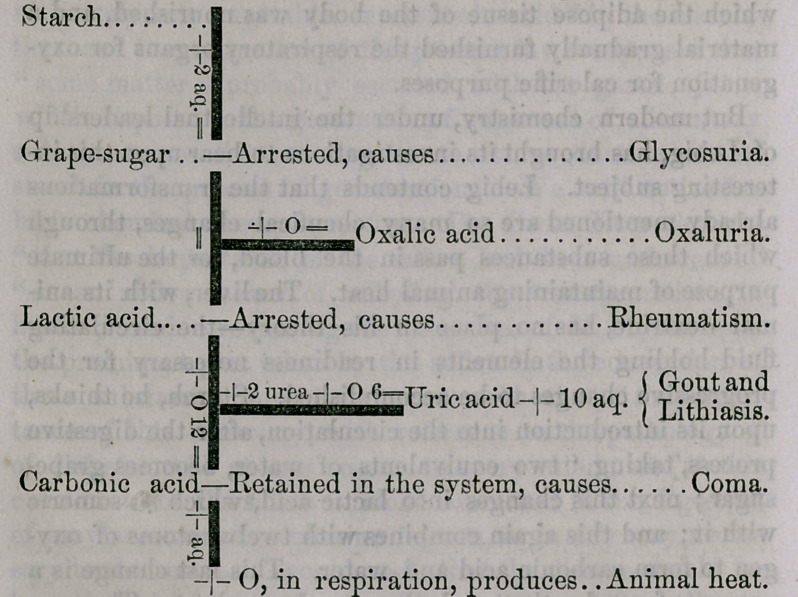# Glycosuria—With the Report of a Case

**Published:** 1866-12

**Authors:** W. T. Goldsmith

**Affiliations:** Atlanta, Ga.


ATLANTA

Medical and Surgical Journal.

NEW SERIES.

VOL. VIIB DECEMBER, 1866. No. 10.

### ORIGINAL COMMUNICATIONS.

### ARTICLE I.

*Glycosuria—with the Report of a Case.* By W. T. GOLDSMITH, M. D., of Atlanta, Ga.

During the month of February, of the present year, a lady, aged 53, of a corpulent habit, sought my advice for what she styled "liver complaint." I found her suffering from marked symptoms of disordered digestion, such as general dyspeptic lassitude after meals, gastrodynia, oppression around the præcordia, sour eructations, and constipation. In early life she had been much troubled by a distressing form of dyspepsia, thought to have been caused by functional disturbance of the liver. In the present instance her rest had been interrupted from inability to sleep, and she seemed to be laboring under general physical and mental depression. Viewing the symptoms as declaratory of dyspepsia, with derangement of the secretory functions of the gastro-intestinal glandular systems, I ordered a cathartic for the relief of the constipation, and when obtained, to be followed by fractional doses of mercury, with an alkali to neutralize the acidity of the stomach. In a few days, the gums having become slightly touched by the mercury, the symptoms disappeared, and the patient was regarded, if not cured, relieved.

A few weeks elapsed, when my assistance was again requested, in consequence of a renewal of the dyspeptic symptoms, but in a modified form. The appetite was good, and improving; there was no distress from pyrosis; no sensation of oppressive weight at the epigastrium; but the constipation was aggravated, and the patient tormented by a violent and insatiable thirst, which was unappeased by the most copious drinks. The tongue was coated white, the skin was harsh and dry, but the pulse natural. Upon inquiry, I learned that much inconvenience was felt from the almost constant desire to void the urine, and that the quantity discharged was unusually large.

Regarding the case as one of probable glycosuria, I requested an examination of the urine, which was found abundant in saccharine matter—so much so as to be plainly perceptible to the taste. The patient was, previous to the first attack, remarkably robust, and had enjoyed almost uninterrupted health since her dyspeptic troubles of early life. When, after the first attack, the most distressing symptoms had disappeared, the appetite had not only remained good, but had increased, notwithstanding the evident marks of emaciation and the consumption, by her, of a larger amount of ingesta. No cause, save that of mental distress, could be assigned as having given rise to the disease.

The diagnosis of glycosuria having thus been clearly made out, the dyspeptic symptoms merited only secondary consideration in the face of the appalling nature of the graver malady; and the treatment sought to be inaugurated was to be based upon strictly physiological principles, to wit: the prevention of the introduction, as far as possible, of sugar into the circulation, by excluding from the dietary all amylaceous and saccharine ingesta, while the distressing dyspeptic symptoms were to be palliated, to the utmost, without loosing sight of the more important indication.

The investigations of McGregor, Mialhe, and others, prove, conclusively, that these substances undergo a series of transformations during the digestive process, eventuating in the formation of sugar, which is discoverable in the portal blood. Bernard supposed that sugar was formed in the hepatic cappillaries, from the portal blood. The fact is, however, established, that sugar is formed in the liver during disease. Why it is that saccharine matter is formed by the liver and poured into the circulation in a state of disease, is impossible to say, as we are in almost total ignorance of the destiny of the animal dextrine, in health. The conversion of this substance into sugar is, emphatically, the result of morbid causes; and in our ignorance of any direct method by which its formation may be checked, or arrested, in the liver, or by that portion of the nervous system controlling it, the indirect method is resorted to of withdrawing every article from the dietary from which it is found capable of being formed. No weapon yet drawn from the armory of the materia medica has been found powerful enough to reach the disorder in its stronghold, and, with the single exception of dietetic management, no important advancement has been made in the treatment of this formidable malady. Hopeless, almost, in my expectation of affording permanent relief—the lady having been made aware of the fearful character of her disease—the treatment recommended by Dr. Prout was thought the best to be employed. Accepting his classification of alimentary ingesta as sufficient for all practical purposes, one of the indications to be met, as above mentioned, was to exclude, as far as practicable, all amylaceous and saccharine substances as articles of food. The patient was, therefore, restricted, as nearly as possible, to animal diet: and, however severe, as a test of endurance, was its adoption, it is recorded, with pleasure, to the patient's credit, of its faithful observance. McGregor had, as has been stated, by a series of well directed experiments, discovered the important fact of the conversion of all amylaceous and saccharine articles of food into diastase; and Dr. Owen Rees found the saliva capable of effecting a similar transformation. The theory, that in glycosuria the diastase so formed was arrested in its course of transformation in the circulation to carbonic acid, and the blood thus surcharged with saccharine matter, has been somewhat shaken by recent investigations. The fact seems to be that the diastase is arrested in the liver, and incorporated into the substance of that organ as animal dextrine. But, whatever theory be adopted, the indication of excluding such articles from the diet list is clearly evident from the results of such treatment; for the fact is unquestionable, that no other plan has ever succeeded in effecting a cure in this disorder: and until we discover the means of controlling the formation of sugar in the body, this treatment presents the only hope of combatting it successfully.

The patient was prohibited from using all articles containing starch, to wit: rice, potatoes, bread, turnips, beans, and articles of this class; all fruits; all articles of diet into which sugar entered, as pastries, etc.; also, the livers of animals, because containing animal dextrine. All kinds of animal diet were to be used: meats, wild and domestic, eggs, butter, oil, broths, milk (though doubtful, as containing saccharine matter), soups, and jellies (without sugar) were to be used in any quantity, and prepared in any way suited to the taste. Cabbage and turnip salad being found to contain but little, if any amylaceous matter, was to be used without restriction. The only form of bread permitted was buckwheat cakes, in moderation, at breakfast; to be eaten with the largest quantity of butter possible.

For the relief of the constipation small portions of ipecac and rhubarb, combined into a pill, were to be taken after each meal. The most prominent symptom—thirst—in the case, was to be relieved. Dr. Prout's suggestion of employing tepid drinks to the exclusion, as far as possible, of all others, was adopted, and phosphoric acid, as recommended by Dr. Latham, used. Both were unavailing, and finally the patient was put upon a decoction of uva ursi, and this, in the end, abandoned. She was, however, restricted, as far as the utmost self-control would allow, in the use of all drinks. Thirst, of all the symptoms of glycosuria, is the most distressing. It depends, no doubt, upon the presence of sugar in the circulation; for this, like every other crystalloid substance, when present in the blood, imperatively demands an amount of water sufficient for its dissolution and elimination from the body. The presence of such a substance permanently into the blood produces, among other effects, a dehydration and disintegration of the tissues which no amount of water introduced into the system can prevent: for it has been proven, by the experiments of Genth, Bocker, and others, that disintegration of the tissues is accelerated by simple transudation of water through the body, and the resulting waste can only be compensated by an increased supply of food. Thirst, therefore, as a symptom of glycosuria, is never removed so long as the disease lasts, disappearing only when the disorder, upon which it is dependent, is cured.

In order to fortify the system against the rapidly progressing emaciation, iron by hydrogen, and cod-liver oil were administered: the former in doses of grains vi, per diem, and the latter to the full extent of stomach toleration.

Emaciation, so characteristic of glycosuria, seems to depend upon several causes: upon impairment of primary digestive assimilation; upon derangement of the secondary assimilative forces; from dehydration and disintegration of the tissues from saccharine matter in the blood, and the water necessary for its dissolution; from the transformation of the liver dextrine into glucose; from the conversion of the adipose tissue, by the liver, into sugar; and, probably, from the interruption of the function of the liver as a blood-making organ.

The improvement, under the treatment employed, in the quantity of sugar in the urine, was immediately perceptible; but the thirst, the emaciation, and the immoderate appetite continued for a length of time. After the expiration of nearly three months, a gradual improvement in every symptom was effected: the constipation had been removed; the thirst had abated; the strength and flesh had increased; the dry and harsh skin had softened; and the urine freed from sugar. The treatment was still continued, and a gradual resumption of the former diet permitted; so that after, the expiration of from four to five months, the lady had returned to her usual dietary habits, and was relieved from all medication.

The favorable termination of the case in health, corroborates the assertions of those most acquainted with the disease, that the prognosis in the corpulent and the aged, is not so grave as in the young, the slender, and delicate. The prognosis is, however, in all cases, highly unfavorable. Glycosuria in persons under twenty years of age is uniformly fatal. Diabetic urine may attend those advanced in years for a long period of time without making material inroads upon the constitution. M. Dechambre thought it was a normal result of old age. The disease, in a majority of cases, is decidedly a chronic disorder; and in inverse ratio to the length of time the symptoms take to complete the full development of the malady, will be the mildness or severity of the disorder—suddenness of attack being less formidable than those cases making almost imperceptible progress, and extending over a considerable period of time.

It is but proper to add, that the density of the urine was not ascertained at any period during the treatment of the case. A specific gravity bottle, or a urinometer could not be obtained. But as the specific gravity of diabetic urine is always increased, depending solely upon the saccharine matter contained, the information revealed by the urinometer is only important in long and protracted cases, where it is desirable to note the quantity of sugar held in solution from day to day.

After the favorable termination of the case, a small tumor was discovered growing rapidly on the cheek under the right eye. It was immediately removed by excision, and the wound cauterized with nitrate of silver. In another month it had returned, growing more rapidly, and it was again removed by excision, with a quantity of the adjacent healthy tissue. Both the parents of this lady had cancer—the father having died with it. The mother still lives, likely, however, to meet the same fate. It was the desire of the writer to use De Morgan's chloride of zinc solution for the destruction of cancer cells—should such prove the character of the tumor—but none was obtained. The wound has, however, healed kindly, with no indication of a return of the tumor up to the present writing.

---

In making the above hasty report of a rare disease, at the request of the senior editor of this Journal, it may not be deemed improper, before dismissing the subject, to give a brief résumé of some of the recently developed physiological and pathological facts in regard to so marvelous a disorder as glycosuria. Sugar, as is well known, is not a natural constituent of the blood, like urea, to be discharged by the kidneys. Its presence in the blood of the systemic circulation, does not always indicate a morbid condition, as it is possible to conceive that the liver may fail to effect the necessary conversion of grape-sugar into dextrine, independent of diseased action, in consequence of over-indulgence in the consumption of amylaceous and saccharine ingesta. A modified form of glycosuria occurs frequently as a complication in other diseases; the saccharine matter, however, is small in quantity, appearing only for a short period, unattended by grave symptoms, and innocent in its results. Such instances of saccharine urine occur frequently from the administration of chloroform and ether; after paroxysms of epilepsy, asthma, and whooping-cough, and after the introduction of poisonous substances into the blood, as strichnia and woorali.

But the disease, clinically known as diabetes melitus, or, the better term, glycosuria, is a grave constitutional malady, stamping its effects upon almost every tissue of the body, and having for its starting point a mal-transformation of a mysterious principle imbedded in the hidden recesses of the liver. Just here an important query arises. What is the cause of this fearful malady? When we trace the history of glycosuria from the time when saccharine matter was first detected in the urine; or from the time when McGregor crowned his investigations by the discovery of diastase in the food after digestion; or the more recent researches of M. Mialhe, upon the transformation of all amylaceous and saccharine ingesta into diastase by the action of saliva; or the still later discoveries of Bernard, Schiff, Pavy, and McConnell of the amyloid substance of the liver,—the accredited causes of this disorder have been as numerous as they were wide of the truth. To exhume the speculations of "the fathers" from the tomb of a departed physiology, in this favored age of microscopic research, would be idle and unprofitable. The very obscurities that veiled the disorder, acknowledged on every hand, furnished inviting fields for ingenious theories, and patient investigation was called upon to unfold the secrets of one of nature's most astounding perversions.

With the lights, however, recently thrown upon the cause of glycosuria the conclusion is logically reached, that it is due to a lesion of a particular portion of the nervous system controlling the vascular system of the liver, by which a peculiar ferment of the blood is brought in contact with the amyloid substance of that organ, resulting in the formation of sugar, and a pouring forth of that substance into the circulation. The lesion of the nervous system is the first link in the chain of causation; the second is in the liver itself, from which flow those characteristic symptoms marking the disease, such as functional disturbance of that organ; general emaciation, from faulty nutrition of the tissues; and a train of dyspeptic symptoms so declaratory of an impairment of the assimilative forces. The cause of glycosuria is, therefore, so intimately connected with its pathology, that we will turn our attention to the particular physiology of the liver, latterly taught, bearing upon the subject, that we may the more readily comprehend its perverted action in this malady. Until recently, the most interesting theory, in regard to glycosuria, related to the liver as a great sugar-forming organ; and the results of the investigations of Bernard, in this direction, seemed so conclusive, that all physiologists accorded to him the honor of a great discovery. They were not slow in adopting so fascinating, and withal, so intelligible a theory as that first promulgated by this great physiologist. The first series of experiments of Bernard seemed to establish the fact, that saccharine matter was being constantly formed in the capillaries of the liver, and discharged into the hepatic veins. But even this great master had occasion to modify his first hypothesis. He found that the saccharine matter in the portal veins, in passing through the liver, was transformed, and incorporated into the substance of the organ: from the fact, that the sugar detected in the hepatic veins could not be formed directly from the blood, as this was discovered to take place after the removal ot the viscous from the animal. Whereupon, another addition was made to the first theory, that the liver contained a peculiar substance capable of conversion into sugar: and as this conversion was found to take place in the liver, when removed from the body, a blood ferment was thought to be necessary in order to effect the transformation. The first supposition of Bernard was, in part, based upon truth. The conversion of amylaceous and saccharine ingesta into diastase is unquestionable. Grape-sugar, formed from these articles, was discovered, in the portal circulation, entering "the gate of the liver." It was found beyond, in the hepatic veins. He found, also, that when no diastase could be detected in the intestines or in the tributary veins of the vena portæ, in animals deprived for hours of amylaceous and saccharine food, that sugar was discoverable in the intralobular veins of the liver; and because of the fact, that sugar was detected in these instances, in the portal blood, just before its entrance into the liver, as well as in the hepatic veins, it was conjectured that the formation took place in the hepatic capillaries, and by reflux, passed into the vena portæ, there being no valves in that system to prevent such action.

We have seen that the transformation of the amylaceous and saccharine articles of diet into diastase is unquestionable. It may not be amiss, in this place, to inquire—how is this conversion effected? So far as the stomach is concerned in the process, the prevailing theory was, that "starch in the stomach passes first into dextrine, then into grape-sugar, and ultimately into vegetable, or carbonic acid" in the circulation: and in explanation of this process, it was supposed that "some matter is probably secreted with the gastric juice, which disposed to the generation of sugar out of alimentary substances, as diastase disposes to its production out of starch in the process of germination." We will notice Lebig's chemical theory hereafter. Dr. Owen Rees thought "that the importance of the saliva had been underrated" "as an active means of effecting the transformations in health;" and in reviewing the researches of M. Mialhe upon the prominent part taken by the saliva in the conversion of these substances into dextrine, remarks that, "the importance of this discovery, with reference to the pathology of of diabetes, will at once occur to every mind; and if this constituent of the saliva is alone concerned in the digestion of amylaceous and saccharine ingesta, or in any way necessary to the result, it would seem that the disease should be investigated with relation to the condition of the salivary glands; a point of view in which it has never yet received the attention of pathologists. M. Mialhe, in a paper on diabetes, has stated it as his belief, that saccharine and amylaceous principles are partly assimilated by an action consequent on admixture with the alkaline matters of the blood." Whatever part the saliva may play in effecting the conversion of alimentary substances into diastase, the isolated fact is proven, that such transformation does occur during the digestive process. The further fact seems also established, by recent experiments, that these series of transformations result in the accumulation in the cells of the liver, of that peculiar principle, animal dextrine. That these transformations serve some ulterior purpose in the animal economy, can not be doubted. What further metamorphosis transpires in the laboratory of the liver in health, from the amyloid substance, can not now be determined. Bernard, as already stated, supposed it to be the conversion of this substance into sugar; and he also entertained the opinion, that a further transformation, resulting in fat and lactic acid, occurred, by which the adipose tissue of the body was nourished, and a material gradually furnished the respiratory organs for oxygenation for calorific purposes.

But modern chemistry, under the intellectual leadership of Lebig, has brought its investigations to bear upon this interesting subject. Lebig contends that the transformations already mentioned are so many chemical changes, through which these substances pass in the blood, for the ultimate purpose of maintaining animal heat. The liver, with its animal dextrine, has no place in his theory—the circulating fluid holding the elements in readiness necessary for the progressive changes to be accomplished. Starch, he thinks, upon its introduction into the circulation, after the digestive process, taking "two equivalents of water, becomes grape-sugar; next this changes into lactic acid, which is isomeric with it; and this again combines with twelve atoms of oxygen to form carbonic acid and water. This last change is a process of combustion, and thus produces heat. The oxygen needed for it is absorbed from the air" during the respiratory process. Lebig thought that he discovered in these transformations the true cause of glycosuria, for it is clear if an arrest in the normal direction of these changes should occur at the period of the formation of grape-sugar—giving credence to the theory upon which it is predicated—the blood would become, by such arrest, charged with sugar. It could not be employed usefully for any purpose of the body, and would consequently be discharged through the kidneys. He also thought there was a manifest connection between a number of diseases depending upon either an arrest or perversion of these transformations. Rheumatism was supposed by him to result in consequence of an arrest at the point of the formation of lactic acid. Grape-sugar, by being oxidized, would give oxaluria. Lactic acid, before passing to carbonic acid, if met by two equivalents of urea, and 6 of oxygen equals uric acid, and ten equivalents of water, would result in gout and lithiasis. The following diagram, modified, from Headland, will explain his theory:

Starch.........

-|-2 aq. =

Grape-sugar ..—Arrested, causes..............Glycosuria.

= -|- O = Oxalic acid...........Oxaluria.

Lactic acid....—Arrested, causes............Rheumatism.

-|- O 12 = -|-2 urea -|- O 6 = Uric acid -|- 10 aq. { Gout and Lithiasis.

Carbonic acid—Retained in the system, causes......Coma.

-|- aq.

-|- O, in respiration, produces..Animal heat.

It will be seen, upon examination of the above diagram, that the perpendicular line indicates the healthy changes through which starch is supposed to pass to fulfill its destiny—the production of animal heat.

The arrest or departure from the normal direction of these transformations must result in disease. "Thus, at each of the two transitional stages, we might have one diseased condition produced by an arrest of the process, and another by its deviation. These deviations and stoppages may result from a failure of some natural principle, which is gifted with the control and direction of the series of transformations; or they may simply be traceable to a want of vital energy or nervous force."*

But, reverting to the discoveries of Bernard, Schiff, and others, it appears that the liver not only controls and appropriates the grape-sugar formed from the amylaceous and saccharine articles of food, but that in the absence of these substances (or of grape-sugar in the portal blood), the liver is capable of transforming the oleaginous articles into sugar. This seems always to be the case in fatal glycosuria. It is equally certain that this wonderful organ has the power of converting these articles into fat. Both conversions may take place independent of the presence of the oleaginous,

---

* The catalytic treatment of rheumatism would seem to afford presumptive evidence of the correctness of the chemical theory of Lebig. This disease on all sides, is supposed to be due to an acid condition of the circulating fluid. That this acid is the lactic, seems to be proven independent of the theory, from its having been discovered, excreted occasionally, by the skin, in arthritic disorders. Should future researches demonstrate its certainty, the formation of the acid upon Lebig's theory, would still be far from being established. The catalytic treatment is an isolated fact, separate and distinct from the theory. The great catalytics in rheumatism are, colchicum, nitro-hydrochloric acid and lemon juice. Nitro-hydrochloric acid is, perhaps, the most powerful oxidizing agent known. Given in rheumatism, it is supposed to effect, by its oxidizing power, a speedy conversion into carbonic acid, which, but for the rapid generation of lactic acid in the system, would promptly arrest the disease. For the same reason lemon juice was found highly valuable by Dr. Rees in rheumatism. Its remedial action evidently depends upon its citric acid. By contrasting the formulars of lactic and citric acids, it will be seen that the citric acid introduced into the blood of a rheumatic, would exercise an oxidizing power, being a carrier of oxygen, and so expedite lactic acid towards its transformation into carbonic acid. Their formulars stand:

Lactic acid, C 6 H 5 O 5 -|- H O.

Citric acid, C 12 H 5 O 11 -|- 3 H O.

However apparently paradoxical, chemists will hardly fail to discover the elements of sound doctrine in the above statement.

The restorative treatment, in rheumatism, by the introduction of alkalies into the blood, however seemingly opposed to the principles of the catalytic, is not so in fact. They are, perhaps, equally valuable as remedial agents in this disease. Wohler has proven that a "vegetable acid becomes oxidized when given in combination with an alkali. So that if we introduce into rheumatic blood a free alkali, a lactate of potash, or of soda will be formed; this may then be enabled to oxidize into a carbonate, and the natural process be completed."

The whole subject is replete with interest, and will, doubtless, receive, at no distant dav, that attention for its more perfect elucidation, its great importance so manifestly demands.

amylaceous, or saccharine articles in the food of the animal—a fact due, in all probability, to the animal dextrine in the liver.

Other physiologists, not accepting in full the recently improved theory of Bernard, have advanced a step further in their investigations. Bernard, although capable of demonstrating, by isolation, the existence of a particular substance termed liver dextrine as present in the liver, failed to fix its precise seat in the organ. It was thought to be diffused throughout the substance of the liver, and its existence demonstrated, not by being detected in the organ, but by isolation from it. Liver dextrine is very abundant in the oyster. It can be easily procured, and in great purity from "the large fawn-colored mass," which is the liver of this mollusk. It may be obtained by the following method: Take a fresh oyster, and holding it over a small vessel of boiling water, clip off, with a pair of scissors, the dark-colored portion, letting it drop in the water. The mass becomes hardened; it is dried and pulverized; after which it is mixed with a small quantity of water, and boiled, and then filtered, and poured into five times its bulk of strong alcohol. A large quantity of a snow-white substance is precipitated, which is the amyloid substance, or animal, or liver dextrine. It is an inodorous substance, dissolving freely in water, and while the salts of copper are not reduced by it, nor will it ferment with yeast, it is transformed readily into glucose, by contact with warm saliva, pancreatic juice, diastase or fresh blood. It is worthy of remark, that although its apparent chemical composition is almost identical with vegetable dextrine, its reaction is not exactly similar. Like vegetable dextrine, it yields a deep wine red coloration with iodine. It is converted into glucose, as stated, by contact with fresh blood, which is not the case with either starch or dextrine. This singular property of the amyloid substance is its most important feature, in its relation to glycosuria, and suggested a series of experiments which resulted in the discovery of a peculiar principle circulating in the blood, called a ferment, which, when brought in contact with the liver dextrine, effects the transformation of that substance into sugar. Schiff and Nasse have, by experimentation, discovered the exact location and physical condition of the amyloid substance in the liver: the former in the livers of frogs; the latter in those of warm-blooded animals. It is found, not dissolved in the hepatic tissue, but in granules, precisely as it occurs with starch, collected in separate vesicles in the liver cells, which, besides the one or two nucleii and fatty globules, contain an immense number of these pale, minute vesicles, within which the amyloid substance is accumulated.

For the better understanding of the exact location in the liver, of this substance, it may not be altogether improper to advert, for a moment, to the minute anatomy of the organ as taught by late physiologists. The vascular arrangement of the liver is well understood. The portal vein entering the transverse fissure, at the base of the organ (in common with the hepatic artery and duct), subdervides until each lobule is more or less surrounded by a venus plexus derived from this source. The cappillaries of the hepatic artery inosculate with those of this plexus—never with those of the intra-lobular veins. From this net-work of blood-vessels of the portal venous system, capillaries radiate towards the centre of the lobule, and are met by the capillaries of the intra-lobular vein, radiating towards the periphery of the lobule. The cellular structure is also found radiating from the centre to the circumference of each lobule, lying between the meshes of the vascular net-work. Microscopic anatomists were long undetermined as to the disposition and arrangement of these cells in the lobules. Kolliker, Handfield Jones, and Dr. Carpenter, thought they were not invested by a basement membrane, but placed "end to end, forming solid cylinders," having no immediate communication with the bile ducts, which were supposed to jut up, by cecal extremities, upon the peripheries of the lobules, while the biliary secretion was transmitted, by transudition, from cell to cell, passing, finally, into the biliary ducts. From analogical deductions, other anatomists thought the cells must be enveloped by a basement membrane; and the fact was ultimately proven to exist by the experiments of Prof. Beale. He injected this membrane, forming a tubular structure surrounding the cells, and found the injection to pass around and enclose them. These cells compose, chiefly, the substance of the liver, and are those from which the bile is elaborated. The secretion is first poured into the interior of the tubular membrane, and then into the biliary ducts, which are continuous with the tubular structure. These cells, according to Todd and Bowman, vary from one thousandth to two thousandths of an inch in diameter. Each cell contains one or two nucleii, with numerous fatty granules, and from the microscopic researches of Schiff and Nasse, the minute, pale vesicles, in which is accumulated the amyloid substance.

When the liver of a recently killed animal is left to itself in a warm place, it will be soon filled with sugar. This occurs when the animal has been deprived of all amylaceous and saccharine ingesta. Should it then be thoroughly washed of its saccharine matter, and left in a warm place, as before, it will again be filled with sugar; and this process of transformation will continue until the liver dextrine shall cease to exist. This process is of easy demonstration in the liver of the oyster; and has also been found to take place in the livers of warm-blooded animals. It appears evident, therefore, that a considerable quantity of amyloid substance exists in the liver, accumulated in the cells of the organ, and that the peculiar ferment of the blood is in close proximity to the animal dextrine, in health, and is brought into immediate contact with it, after death.

The animal dextrine, as has been remarked, has been found in the livers of all animals, in health, when recently killed. But it readily disappears under a variety of circumstances and diseased conditions. It is rarely, if ever, detected in the human liver. It could only be found in the livers of those dying suddenly, as it disappears under the influence of disease, during protracted illness.

The peculiar ferment of the blood, which has been supposed necessary for the conversion of liver dextrine into sugar, has never been demonstrated to exist by isolation. The fact, however, of its existence can not be doubted. This is proven by the fact already noticed, that the process of transformation of the dextrine into sugar continues after death, when all vital activity has ceased. This process continues even after every trace of sugar has again and again been washed from the organ. It has been further proven by the remarkable fact in regard to the periodical absence of this ferment, in the blood of frogs. According to Schiff, it has been found to disappear from these animals "during the recent half of winter and the early spring months. This occurs as a regular event in the annual changes which these bactracians undergo. During this interval, the liver is as full as usual of amyloid substance; but no spontaneous production of sugar occurs, when the organ is abandoned to itself in a warm place: and artificial glycosuria cannot be engendered in such animals. When, however, the blood of another animal, which is not in this peculiar condition, is injected into the bodies of frogs, or applied to their livers, the usual production of sugar takes place rapidly."

It has not been positively determined what special mission animal dextrine subserves in healthy life. The liver is supposed to be, by some very eminent physiologists, a blood-forming organ. Weber discovered in the liver of the embryo abundant generation of blood-corpuscles—a fact confirmed by Kolliker. Weber believed that the liver separated and appropriated in its substance, a material from the blood, from which blood-corpuscles were generated. This seems fully susceptible of proof in the liver of the embryo chick—blood-corpuscles being elaborated from the yelk-globules in the liver, and poured into the circulation. A similar metamorphosis, as in that of the chick, was observed by Weber to occur in the frog in the spring of the year. In view of the facts bearing upon the subject, Todd and Bowman suggest the query, "whether the liver may not afford a source of supply of blood-corpuscles, or contribute to the production of hæmatine in adult life?" In response to the inquiry suggested, they answered: "It has often struck us that this question might be answered in the affirmative, while observing cases in which the process of the formation of blood seemed greatly perverted, when no organic disease could be detected beyond some degree of enlargement of the liver. Patients suffering in this way are pale, as if from loss of blood, although no such loss had been experienced: their nutrition is enfeebled, digestion impaired, and there is slight yellowness of the complexion, as in cases of hepatic disease; and, after death no lesion is discoverable, but slight enlargement of the liver."

It is known "that the venous blood of the spleen passes along with that from the stomach and intestines through the liver. Recent researches of Kolliker and Ecker offer some explanation of this fact, and at the same time of the relation between hæmatine and the coloring matter of bile, as well as between the office of the liver, and the generation of the red particles of the blood. It would appear from these researches," "that the red blood-corpuscles undergo decay in the red substance of the spleen, giving up their hæmatine, in an altered form to the portal blood, from which it may not improbably, as Kolliker conjectures, pass into the bile-cells to form, and to be eliminated as the biliary coloring matter; and, perhaps, also to contribute to supply hæmatine to new blood-cells developed in the liver."

Should these conjectures prove to be correct, it would appear that the liver and spleen have functions somewhat in common, as blood-forming organs. They have the glycogenic power in common, as the spleen has been found to be, to a limited extent, a sugar-forming organ. The corpora amylacea of the spleen were, by eminent physiologists, supposed to be the seat of the substance capable of transformation into sugar. Salisbury, however, in a series of late investigations, asserts that these bodies are neither allied to starch or cellulose. But that the spleen is a sugar-forming organ, is admitted by him in the following language: "When the spleen of the common fowl is set aside, exposed to conditions which induce decay"—just as occurs in experiments with the liver—"in connection with the decay which takes place, alcoholic fermentation sets in, which develops *torula cells and filaments* in considerable quantities, producing in a short time a white mould, or dust, of cells and filaments over the whole surface. These torulæ cells are smaller than those developed in fermenting liver, but of the same general character. The development of these cells in the decaying spleen is an indication of the presence of glycogenic matter, or sugar, in some form, and which is probably generated by it."

The spleen is found also to elaborate from the myoline cells, cholesterin, which, being conveyed by the portal blood to the lobular structure of the liver, is secreted as one of the constituents of the bile. A late writer affirms "that a careful examination of the substance of the liver has determined the fact, that cholesterin and serolin are not formed in the cells of this organ, but simply secreted by the biliary apparatus." These facts, which may seem irrelevent to the subject, are introduced in order to show the intimate physiological connection between the liver and the spleen. They would seem to give some foundation to the opinion that the amyloid substance of the liver may be in some way concerned in the perfection of the blood and the secretion of bile in health; and that one of the results of glycosuria in producing rapid emaciation, may be due, in part, to the suspension or interruption of this function of the liver.

McConnell, "in his recent memoir on the functions of the liver, brings forward some facts and considerations of great weight in support of his view, that the great destiny of liver dextrine is to unite with nitrogen (set free by the dis-assimilation of fibrin and a portion of the albumen of the portal blood) so as to constitute a new protein compound resembling cassin, which is being constantly poured into the circulation through the hepatic veins."

Bernard was of the opinion that a constant transformation of the amyloid substance into sugar was one of the functional duties of the liver: that while this organ was secreting bile, and pouring it into the biliary ducts in one direction, the dextrine was being converted into sugar, and poured into the intra-lobular veins in the other. But, however ingenious and seemingly conclusive the glycogenic theory of Bernard, and the experiments upon which it was based, other investigators, as Prof. Roberts and McConnell, have proven that such transformation takes place only during disease, or in the newly killed animal, and never in health. The glycogenic theory rested mainly upon the fact, that in recently killed animals the blood of the hepatic veins contained a larger and richer supply of sugar than that of any other part of the body. Pavy discovered that the appearance of sugar in the hepatic veins resulted from the rapid transformation of the dextrine, in consequence of the injuries sustained by the animal during the performance of the experiment. He changed the mode of operating, so as to avoid, as far as possible, all disturbing causes; and hepatic blood so procured contained no more sugar than other and remote portions of the circulation. McConnell repeated and improved these experiments, obtaining "results which do not seem to admit a possibility of doubt, that amyloid substance is not converted into sugar during healthy life." Roberts has also repeated these experiments, and has never been able to detect the slightest trace of saccharine matter in the livers of frogs and oysters while in health.

From the above review of this important part of the physiology of the liver, we are somewhat prepared to inquire into the pathology of glycosuria. We will give, therefore, what seems to be the most plausible theory, in accordance with the facts, as to the pathological condition necessary for the production of this disease. We have seen from the experiments of Pavy, McConnell, and others, that the amyloid substance in the liver is never transformed into sugar during health. This substance is always found in the liver; and the peculiar blood-ferment is never, under favorable circumstances, absent. We find, then, a principle in the blood, carried perpetually through the liver, in close proximity to its amyloid substance, capable of instantly effecting a conversion of its dextrine into sugar; and yet, this transformation never occurs except as the result of disease, some abnormal condition, or after death.

The query immediately suggests itself: What pathological condition is necessary to bring in contact the blood-ferment and the amyloid substance? The solution of the question would unfold the whole pathology of glycosuria. It can not be answered fully; but some glimmerings of the truth have been seen, and the day may not be far distant when it shall be revealed in all its grandeur to the earnest inquirer. The obscurities that, in many particulars, envelope the physiology of the liver, is an impassable barrier to the study of the pathology of a disease so intimately connected with the healthy action of the organ. Could we unfold the varied processes and transformations effected in this wonder-working viscus, and that still more wonderful tissue, the circulating fluid, we might hope to unravel the secrets that now shroud the subject in mystery.

The experiments of physiologists, in the production of artificial glycosuria in animals, have thrown a flood of light upon the subject. Glucose urine may be engendered in various ways. M. Reynoso found the urine saccharine in persons who had been under the influence of chloroform and ether: he also discovered it in the urine of patients laboring under "tuberculosis, pneumonia, chronic bronchitis, asthma, asphyxia, hysteria, and epilepsy; and after the use of various medicines, as opium and other narcotics, quinia, mercury, etc." It is also caused by impeding the respiration; by poisoning with strychnia and woorali; by tying the afferent veins of the kidneys; by thrusting needles in the liver, and by injecting acids into the portal veins. Reynoso "is disposed to think that whatever interferes with the respiratory process, whether directly, as diseases of the chest, or indirectly, through the nervous centers, as nervous affections and narcotics, may produce this effect." Diabetic urine was noticed by Becqurel in cerebral, spinal, and hepatic diseases. Organic diseases of the brain and spinal cord have recently been shown to occasion glycosuria. Cerebral hemiplegia, in a case of Pavy's, excited the disease. Dr. Gull discovered sugar in the urine of a patient after having an apoplectic fit. Fritz has collected a number of cases occurring after cerebral softening, tumors of the pia mater, general paralysis, and myelitis. Numerous cases have occurred, resulting from external injuries of the brain and other parts: these injuries consisting in blows and falls on the forehead, occiput, vertex, fractures of the vertebræ, etc. Some of these injuries induced permanent glycosuria; others were transient, subsiding with the cerebral or other symptoms. These apparent multifarious causes of the disease afford special interest, as bearing on the discoveries of Bernard, Schiff, and Pavy, in the production of artificial glycosuria in animals by cutting and puncturing different portions of the nervous system; and these experiments seem to develop the fact, that all injuries sustained by the brain and spinal cord, harmonize with all other causes producing glycosuria, and that they depend upon irritation being applied, directly or indirectly, to some portion of the nervous system controlling the vascular system of the liver. In all diseases impeding the respiration, or when narcotism or anæsthesia is produced by medicines, the irritation is conveyed through the pneumogastric nerves, and reflected to the liver; strychnia acts directly upon the spinal cord, involving the nerve fibres proceeding to the liver. Diseases and injuries of the brain and spinal cord, either by direct irritation or reflex action, influence the nerve-arrangement of the organ, and so, however varied the points of departure, the focus of the disease must be looked for in some centre of the nervous system.

Bernard, with wonderful skill, punctured the floor of the fourth ventricle of the brain. When a particular spot of the floor was irritated, a copious secretion of urine was immediately set up. When another portion was wounded, midway between the origins of the auditory nerves, an instant discharge of sugar was poured into the circulation, and the urine became saccharine. The liver would seem, then, to be "under the control of a distinct nerve-arrangement, with a local centre in its neighborhood (probably the cœliac ganglion), and upward prolongations, by the sympathetic and spinal cord, into the cerebral centres. The separate threads of this communication are, in the lower parts of their course, placed widely apart; but they approach in the spinal cord, and in the floor of the fourth ventricle are collected into a close bundle before their final dispersion into the cerebral hemispheres." Irritation, applied to any portion of this nervous route, causes, at once, the immediate production of glycosuria. The great difficulty of puncturing the precise spot in the ventricle induced Schiff to cut the entire cord, at the points of origin of the brachial nerves, which was always attended by the appearance of sugar in the urine. It is not difficult to conceive that irritation, congestion, inflammation, or pressure of the cœliac ganglion, might produce a similar result. Such, no doubt, is the case.

The vascular system of the liver, like that of every other part, is under the control of a special portion of the nervous system. We have seen that the nerve-fibres, starting from the fourth ventricle of the brain, pass to the liver and kidneys. This distribution seems to be exclusively to the coats of the blood-vessels of these organs. The muscular tissue constituting the circular and longitudinal coats of these blood-vessels, are, by this nerve-arrangement (nervi vasi motores), provided for an active contraction of their calibres. The negative power of expansion is equally active under the pressure of the blood, when the contractile tissue is paralyzed, and by the elasticity of their coats. In polyuric subjects the renal vessels seem to be paralyzed, and in glycosuria the muscular fibres, both circular and longitudinal, are in a paralytic condition. In the one case, copious diuresis ensues; in the other, instant contact of the blood-ferment with the amyloid substance occurs, and sugar discharged into the hepatic veins. All the diseased conditions, however various, resulting in glycosuria, can be accounted for upon this theory, which accords both with experiments upon animals and clinical experience.

Why it is that paralysis of the contractile tissue of the hepatic blood-vessels brings in contact the blood-ferment and the liver dextrine, has not been determined. It has been thought to be consequent upon the hyperæmia induced, and, possibly, by a saturation, more or less of the cellular structure, with the blood-ferment, and to a dissolution, to some extent, of the cells containing the amyloid substance; or, to loss of function of the vascular system paralyzed, controlling the secerning office of the liver, and disturbing the normal constituents of the cells. But great hyperæmia may exist in the liver without the appearance of sugar in the urine; and, even in cases of temporary glycosuria, upon the supervention of positive pyrexia, the glycogenic condition is suspended. We are, however, justified in asserting, that the only known pathological condition for the production of glycosuria is to be found in a lesion of a particular portion of the nervous system controlling the vascular system of the liver; and, although we may not, in every case, discover the precise spot involved, the fact is nevertheless conclusive, that the effect upon the contractile tissue of the hepatic blood-vessels is always the same—i. e., paralysis of the muscular coats, followed, perhaps, by hyperæmia.

---

Notwithstanding the length of this article, we do not feel that we should close without referring to the methods employed for the detection of sugar in the urine. When glycosuria is well developed, the urine may, for all practical purposes, be considered a solution of grape-sugar in water—being chemically identical with this substance, or glucose. The other constituents are vastly disproportioned in comparison to their normal proportions, in the urinary discharge. When sugar is present in the urine it may be easily detected; but the usual methods of procedure—from the want of the necessary cautions, or from a want of information as to the manner of their application—are liable to many errors. Saccharine matter has often appeared to be present, when tested for, when none existed; and, on the other hand, a large proportion of sugar may be in solution in the urine, when the tests employed fail to reveal it.

It is our purpose, then, to examine, briefly, some of the usual methods of sugar testing, and show why, as commonly employed, they are subject to fallacies. "When the flow is considerable" in glycosuria "the urine has a very pale straw tint, and a peculiarly bright aspect. It speedily becomes opalescent when exposed to the warm air, and in a few hours ferments, with abundant disengagement of gas, and production of sporules and filaments of the yeast plant. These latter form a white flour-like deposit in diabetic urine, after it has been kept awhile." The appearance of the yeast plant in the urine, is not, however, conclusive of the presence of sugar in the secretion: for *torulæ* are not confined, exclusively, to saccharine urine. The yeast plant may grow to full fructification in the urine without the most delicate tests being sufficient for the detection in it of sugar. This remarkable vegetation (*torula cerevisiæ*) undergoes almost the same phases of development as that of the mould fungus (*Penicilium glaucum*). They are both found running through three phases to the period of full development: the first phase is in oval cells or sporules; the second, in interlacing fibres or thallus; the third, the development of the plant growing in the air, or, ariel fructification. It is only in the third and last phase that the yeast plant can be distinguished from the mould fungus. In the yeast fungus the stalk—in the phase of ariel fructification—terminates in a spherical head full of sporules: the mould fungus in the same phase has a tuft of branches proceeding from the stalk. So similar are they in their features, that they are not of easy discrimination. It will be seen, therefore, that the reliability of *torulæ*, as indicative of the certainty of the saccharine quality of the urine, is far from being true; yet, in some of our text-books, it is stated to be so, without qualification.

The boiling of liquor potassæ with urine—called Moore's test—cannot be relied upon in every case, in revealing sugar when present in the urine. It is wanting in delicacy, as it requires a grain and a half of sugar to the ounce of urine, before it can be made available. A patient may have incipient glycosuria, and this test fail to discover the fact. The fermentation test is inferior to that of Moore's, as it requires two grains and a half of sugar to the ounce of urine, before detection is possible.

Reduction tests are by far the most delicate and reliable. They are, however, frequently fallacious, from a want of care, and a knowledge of the proper method of proceeding. When employed as commonly recommended, Trommer's test is as unreliable as those referred to above. The urine, according to many of our text-books, is first to be boiled, and the solution of copper added: while the solution of copper, not being prepared according to the proper standard, is either above or below the proper strength. The following plan for sugar testing with copper, is the one adopted by an eminent English chemist, and superior to all others known to the writer: "Pour some of the prepared test-liquor* in a narrow test-tube, to the depth of three quarters of an inch; heat until it begins to boil; then add two or three drops of the suspected urine. If sugar be abundant, a thick, yellowish opacity, and deposit of yellow suboxide are produced (and this changes to a brick-red at once, if the blue color of the test remain dominant). If no such

* The prepared test-liquor, or Fehling's standard copper solution is prepared according to the following formala:

| | | |
|---|---|---|
| Sulphate of copper | gr. | 90½ |
| Neutral tartrate of potash | " | 364 |
| Solution of caustic soda, sp. grav. 1.12 | f. oz. | 4 |

Add water to make exactly six fluid ounces.

reaction ensue, go on adding the urine until a bulk nearly equal to the test employed has been poured in: heat again to ebullition; and, no change occurring, set aside without further boiling. If no milkiness is produced as the mixture cools, the urine may be confidently pronounced free from sugar, for no quantity above a fortieth of a grain per cent. can escape such a search, and any quantity below this, is devoid of clinical significance." "The points of importance in this proceeding, are to boil the test first, and not the urine; and to use an excess of the test."

The volumetrical method, employed by Fehling, with perfect success, consisted in first establishing by experiment, the relation between the amount of the solution of copper, when reduced to a suboxide with the sugar present. He found that 180 parts of grape-sugar decomposed precisely 1246.8 parts of copper solution—or one equivalent of grape-sugar to ten of the solution. His standard solution containing 200 grains, is exactly decomposed by one grain of sugar.

The differential density method is regarded, for convenience and accuracy, as containing more advantages than any other. The principle of this method depends upon a comparison between the specific gravity of glucose urine, and such urine deprived by fermentation with yeast, of its saccharine material. Roberts, in his late superb work, from which the above methods of sugar-testing have been drawn, in reference to this method, says: "The mode of experimenting was: first, to ascertain by the volumetrical analysis," "how much sugar was contained in a certain diabetic urine. The urine was then fermented by means of German yeast—its specific gravity having been previously ascertained. In twenty-four hours after the fermentation had ceased, and the scum had subsided, the density was taken again; and by subtracting this from the density before fermentation, the 'density lost' was ascertained. And it was found that for every grain of sugar contained in an ounce of urine, one degree of specific gravity had been lost. Experiments were multiplied on diabetic urine: corresponding experiments were made with solutions of sugar of known strength, in healthy, non-saccharine urine, and pure water, and the issue of all was to establish the conclusion, that *the number of degrees of 'density lost' indicated as many grains of sugar per fluid ounce*:" truly a beautiful and delicate method of determining the quantity of sugar in diabetic urine.

## Figures and Tables

**Figure f1:**